# Postbiotics Prepared Using *Lactobacillus paracasei* CCFM1224 Prevent Nonalcoholic Fatty Liver Disease by Modulating the Gut Microbiota and Liver Metabolism

**DOI:** 10.3390/ijms232113522

**Published:** 2022-11-04

**Authors:** Zhenghao Pan, Bingyong Mao, Qiuxiang Zhang, Xin Tang, Bo Yang, Jianxin Zhao, Shumao Cui, Hao Zhang

**Affiliations:** 1 State Key Laboratory of Food Science and Technology, Jiangnan University, Wuxi 214122, China; 2 School of Food Science and Technology, Jiangnan University, Wuxi 214122, China; 3 National Engineering Research Center for Functional Food, Jiangnan University, Wuxi 214122, China

**Keywords:** postbiotics, high-fat diet, NAFLD, gut microbiota, liver metabolome

## Abstract

Postbiotics are rich in a variety of bioactive components, which may have beneficial effects in inhibiting hepatic lipid accumulation. In this study, we investigated the preventive effects of postbiotics (POST) prepared from *Lactobacillus paracasei* on non-alcoholic fatty liver disease (NAFLD). Our results showed that when mice ingested a high-fat diet (HFD) and POST simultaneously, weight gain was slowed, epididymal white fat hypertrophy and insulin resistance were suppressed, serum biochemical indicators related to blood lipid metabolism were improved, and hepatic steatosis and liver inflammation decreased. Bacterial sequencing showed that POST modulated the gut microbiota in HFD mice, increasing the relative abundance of *Akkermansia* and reducing the relative abundance of *Lachnospiraceae NK4A136 group*, *Ruminiclostridium* and *Bilophila*. Spearman’s correlation analysis revealed significant correlations between lipid metabolism parameters and gut microbes. Functional prediction results showed that the regulation of gut microbiota was associated with the improvement of metabolic status. The metabolomic analysis of the liver revealed that POST-regulated liver metabolic pathways, such as glycerophospholipid and ether lipid metabolism, pantothenate and CoA biosynthesis, some parts of amino acid metabolism, and other metabolic pathways. In addition, POST regulated the gene expression in hepatocytes at the mRNA level, thereby regulating lipid metabolism. These findings suggest that POST plays a protective role against NAFLD and may exert its efficacy by modulating the gut microbiota and liver metabolism, and these findings may be applied to related functional foods.

## 1. Introduction

Non-alcoholic fatty liver disease (NAFLD) is a multifactorial condition with multiple pathogeneses, including hepatic lipogenesis regeneration, oxidative stress, insulin resistance (IR), inflammasome activation, and/or fibrogenesis [1]. NAFLD, especially the histological phenotype of nonalcoholic steatohepatitis, can cause severe liver diseases, such as liver cirrhosis and cancer [2]. Increases in high-fat diet (HFD) intake and the resulting obesity show a parallel with the global increase in NAFLD [3]. NAFLD has become a severe health concern worldwide owing to rising obesity rates. While there are several active phase 2 and 3 trials evaluating treatments for NAFLD, no FDA-approved pharmacologic medications are currently available [4]. Owing to the serious harm caused by NAFLD and the lack of mitigation methods, there is an urgent need to explore new methods to prevent and treat NAFLD.

In 2021, the International Scientific Association of Probiotics and Prebiotics (ISAPP) officially defined postbiotics as inactivated bacteria and/or their components that exert a positive effect on the health of the host [5]. A large number of bioactive metabolites, such as organic acids and exopolysaccharides, were found in postbiotic preparations of lactic acid bacteria [6]. Based on the abundant bioactive substances in postbiotics, various studies have recently been undertaken to establish how postbiotics operate and should be utilized [7]. Inactivated probiotics improve insulin resistance (IR) and reduce fat gain in HFD mice, possibly for the amelioration of NAFLD [8]. Bacterial lysates have also been reported to improve glucose metabolism, reduce body and liver lipid levels, reduce hepatic immune cell infiltration, and alleviate NAFLD in HFD mice [9]. Therefore, postbiotics have great potential in the management of NAFLD owing to their richness in bioactive substances and good stability (no live bacteria). 

The hepatic portal system anatomically connects the liver and the gut. There is increasing evidence that the development of NAFLD is closely related to the dysbiosis of the gut microbiota and that the gut and liver are closely linked to metabolic function [10]. In one study, germ-free mice developed symptoms of NAFLD after receiving a fecal transplant in a mouse model of HFD-induced NAFLD [11]. HFD can lead to an imbalance of gut microbiota, such that endotoxins and inflammatory factors produced in the intestine can enter the liver through the gut-liver axis, causing inflammation, IR, and fat metabolism dysfunction in the liver, which in turn induces NAFLD [12]. Numerous studies have shown that probiotics and prebiotics can alleviate NAFLD by modulating gut microbiota dysbiosis [13]. Postbiotics may also have the potential to alleviate NAFLD. However, whether the role of postbiotics is related to the gut microbiota remains to be further investigated. 

Metabolomics can elucidate the mechanism of action of drugs at the metabolic level by systematically identifying and quantifying metabolite levels [14]. Liquid chromatography-mass spectrometry (LC-MS) is regarded as a suitable platform for metabolomic studies as it is a quick metabolomic analysis method [15]. It has been previously discussed how NAFLD causes metabolic pathways to become dysfunctional [16]. The development of NAFLD involves various metabolite changes, and these changes and the mechanisms behind them can be analyzed using LC-MS [17]. Therefore, there is a new method to explore the mechanisms by which substances alleviate NAFLD. Studies have shown that *Lactobacillus paracasei* can regulate liver glycerophospholipid and arachidonic acid metabolism, fatty acid breakdown, and other metabolic pathways, thereby reducing liver lipid accumulation [18]. However, the relationship between the preventive effect of postbiotics prepared using *Lactobacillus paracasei* on NAFLD and liver metabolomics remains to be studied.

In this study, we established a NAFLD mouse model using HFD and explored the preventive effect of postbiotics (POST) prepared using *Lactobacillus paracasei* CCFM1224 on NAFLD. The mechanism of postbiotic protection against NAFLD was investigated by studying liver metabolomics using LC-MS and the gut microbiome using high-throughput sequencing. At the same time, the relationship between the lipid metabolism phenotype and gut microbiota was revealed, which provided a reference for the development of postbiotic products to alleviate NAFLD.

## 2. Results

### 2.1. POST Ameliorated the Obesity-Related Parameters

**Figure 1:**
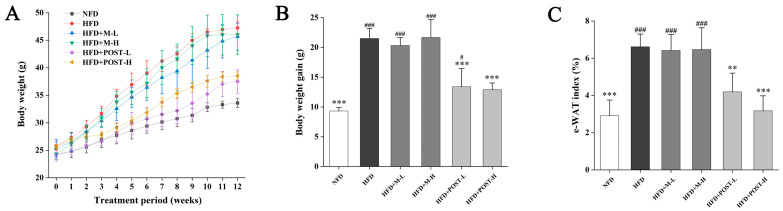

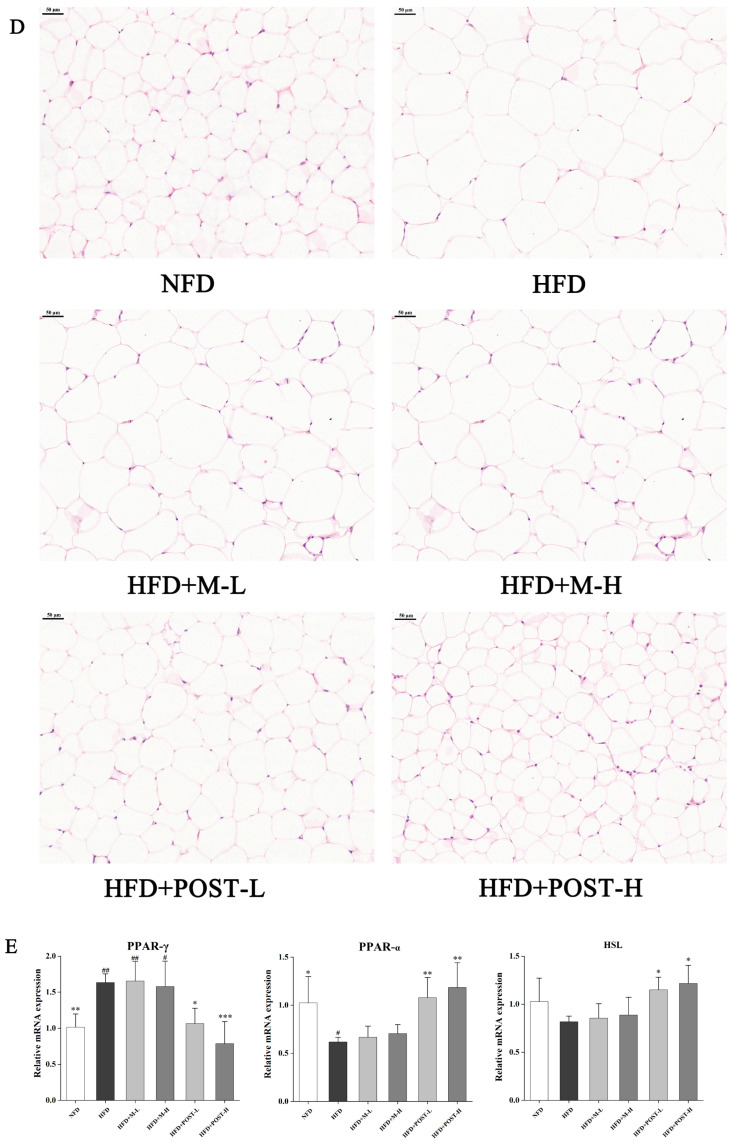
POST ameliorated obesity in HFD mice. (**A**) Weight change over 12 weeks. (**B**) Total weight gain. (**C**) The e-WAT index. (**D**) H&E staining of e-WAT (magnification 200×). (**E**) Relative mRNA expression of PPAR-γ, PPAR-α, HSL in the e-WAT. Compared to HFD, * *p* < 0.05, ** *p* < 0.01, and *** *p* < 0.001; Compared to NFD, # *p* < 0.05, ## *p* < 0.01, and ### *p* < 0.001.

To investigate whether POST prevents HFD-induced obesity, it was administered to HFD mice for 12 weeks. After ingesting the HFD, the mice had markedly increased body weight, and significant epididymal white fat accumulation (e-WAT), whereas the POST intervention significantly suppressed body weight gain (HFD + POST-L vs. HFD: decreased 0.38-fold, *p* < 0.001; HFD + POST-H vs. HFD: decreased 0.40-fold, *p* < 0.001) and e-WAT accumulation (HFD + POST-L vs. HFD: decreased 0.37-fold, *p* < 0.01; HFD + POST-H vs. HFD: decreased 0.52-fold, *p* < 0.001) in HFD mice, while the diameter of e-WAT in POST intervention groups was substantially smaller than that in HFD group (Figure 1A–D). However, the M intervention failed to improve HFD-induced weight gain and e-WAT accumulation (Figure 1A–D). The expression of lipid metabolism genes in e-WAT was further investigated (Figure 1E). HFD significantly promoted the expression of peroxisome proliferator-activated receptor gamma (PPAR-γ) but significantly inhibited the expression of peroxisome proliferator-activated receptor alpha (PPAR-α). However, after POST intervention, the mRNA expression of PPAR-γ (HFD + POST-L vs. HFD: decreased 0.35-fold, *p* = 0.0172; HFD + POST-H vs. HFD: decreased 0.52-fold, *p* < 0.001) was significantly decreased, the mRNA expression of PPAR-α (HFD + POST-L vs. HFD: increased 0.74-fold, *p* = 0.0081; HFD + POST-H vs. HFD: increased 0.92-fold, *p* < 0.001) and hormone-sensitive lipase (HSL) (HFD + POST-L vs. HFD: increased 0.41-fold, *p* = 0.0482; HFD + POST-H vs. HFD: increased 0.49-fold, *p* = 0.0119) was significantly increased.

### 2.2. POST-Attenuated IR and Improved Serum Lipid Parameters

**Figure 2:**
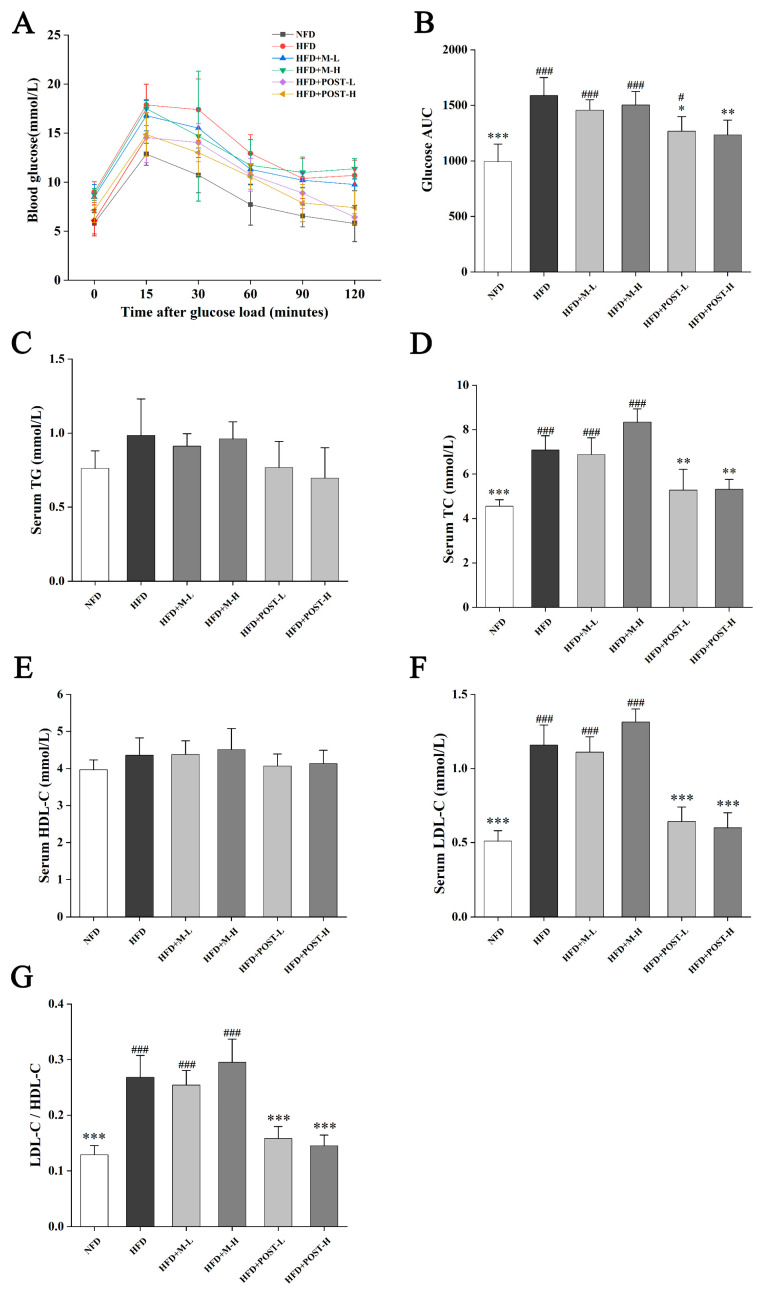
POST improves IR and serum lipid parameters in HFD mice. (**A**) OGTT showed changes in blood glucose at different time points after oral glucose. (**B**) The AUC according to the OGTT curve. (**C**–**F**) Concentrations of serum lipids indices, namely TG, TC, LDL-C and HDL-C. (**G**) LDL-C/HDL-C value. Compared to HFD, * *p* < 0.05, ** *p* < 0.01, and *** *p* < 0.001; Compared to NFD, # *p* < 0.05, and ### *p* < 0.001.

In terms of glucose metabolism regulation, according to the experimental results of OGTT, HFD mice had higher fasting blood glucose and impaired glucose tolerance than NFD mice, but the glucose tolerance of the POST intervention groups increased significantly (Figure 2A). AUC was calculated to confirm this conclusion (Figure 2B). Increased serum lipid levels are the primary features of HFD mice. Therefore, we determined the serum lipid levels in mice to evaluate the efficacy of POST. Compared with that of NFD mice, the serum TC, LDL-C, and LDL-C/HDL-C values of HFD mice significantly increased after the ingestion of HFD. Compared with that of the HFD group, POST intervention significantly reduced the serum TC (HFD + POST-L vs. HFD: decreased 0.26-fold, *p* = 0.0023; HFD + POST-H vs. HFD: decreased 0.25-fold, *p* = 0.0029), LDL-C (HFD + POST-L vs. HFD: decreased 0.45-fold, *p* < 0.001; HFD + POST-H: decreased 0.48-fold, *p* < 0.001), and LDL-C/HDL-C (HFD + POST-L vs. HFD: decreased 0.41-fold, *p* < 0.001; HFD + POST-H: decreased 0.46-fold, *p* < 0.001) values, showing the ability to reduce HFD-induced hyperlipidemia, whereas M intervention had no such effect (Figure 2D,F,G). It is worth noting that the serum TG and HDL-C levels of the mice in each group did not show obvious differences (Figure 2C,E).

### 2.3. POST Prevented Hepatic Steatosis and Dysfunction

**Figure 3:**
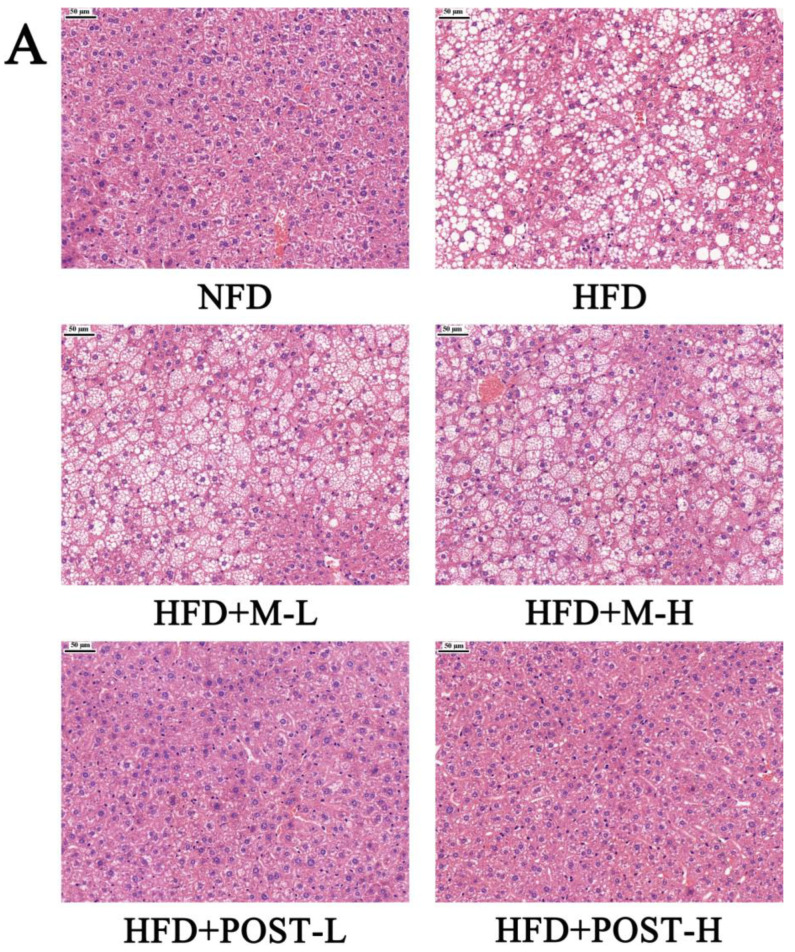

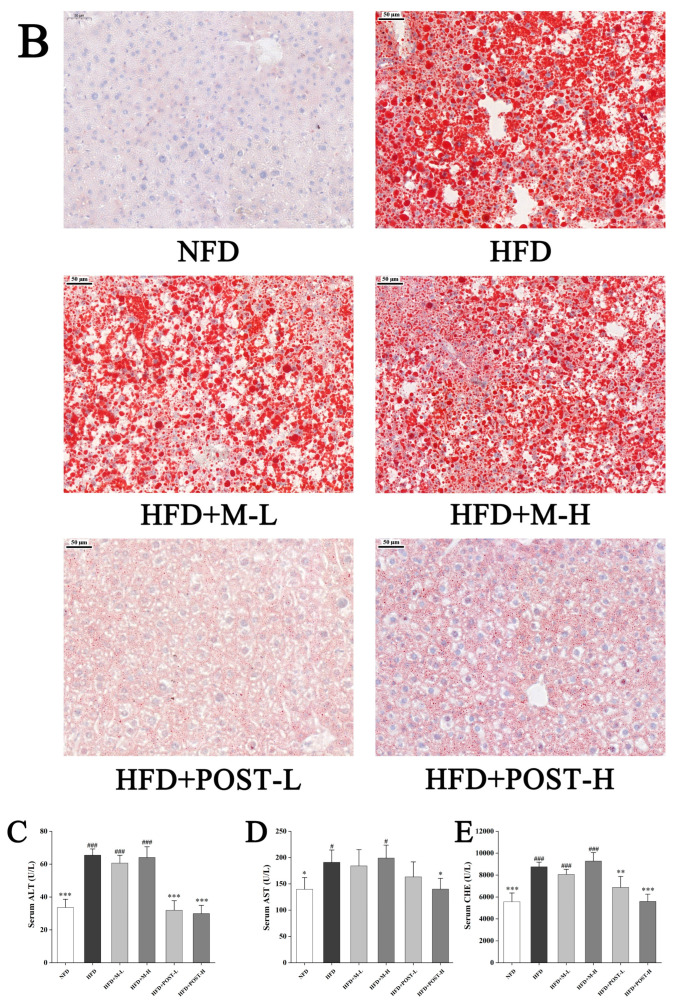
POST improves hepatic steatosis and liver function in HFD mice. (**A**) H&E staining of the liver (magnification 200×). (**B**) Oil Red O staining of the liver (magnification 200×). (**C**–**E**) Serum content of ALT, AST, CHE. Compared to HFD, * *p* < 0.05, ** *p* < 0.01, and *** *p* < 0.001; Compared to NFD, # *p* < 0.05, and ### *p* < 0.001.

In the assessment of NAFLD, H&E and/or Oil Red O staining analysis, which is evaluated by section morphology, is often used. The H&E staining results showed that the liver tissue sections of NFD mice had regular morphology, while the liver tissue sections of HFD mice were filled with a large number of fat vacuoles with severe steatosis (Figure 3A). The results of Oil Red O staining also showed the same trend as H&E staining, with a greater accumulation of lipid droplets in the liver tissue of HFD mice compared to that in NFD mice (Figure 3B). Hepatic steatosis was suppressed in the POST intervention group (Figure 3A,B). Qualitative histopathological analysis of liver sections revealed that mice exhibited significant hepatic steatosis (grade 7) and inflammation (grade 1) after HFD ingestion, while the liver steatosis (HFD + POST-L: grade 1, HFD + POST-H: grade 0) and inflammation (HFD + POST-L: grade 0, HFD + POST-H: grade 0) were significantly ameliorated after the POST intervention (Table 1). Hepatic steatosis is often accompanied by hepatic impairment, and serum liver enzyme levels are sensitive indicators of liver injury. Changes in the levels of liver enzymes (AST, ALT, and CHE) in the serum were evaluated in all groups of mice after the histological observation of liver damage, and the results revealed that the HFD produced a considerable increase in serum liver enzyme levels (Figure 3C–E). Compared with HFD mice, the HFD + POST decreased the serum liver enzyme activities in a dose-dependent manner, namely ALT (HFD + POST-L vs. HFD: decreased 0.51-fold, *p* < 0.001; HFD + POST-H vs. HFD: decreased 0.54-fold, *p* < 0.001), AST (HFD + POST-L vs. HFD: decreased 0.15-fold, *p* > 0.05; HFD + POST-H vs. HFD: decreased 0.26-fold, *p* = 0.0414), and CHE (HFD + POST-L vs. HFD: decreased 0.21-fold, *p* = 0.0043; HFD + POST-H vs. HFD: decreased 0.36-fold, *p* < 0.001) (Figure 3C–E). However, the M treatment failed to ameliorate hepatic steatosis and liver injury in HFD mice (Figure 3A–E).

### 2.4. POST Attenuated HFD-Induced Hepatic Inflammation

**Figure 4:**
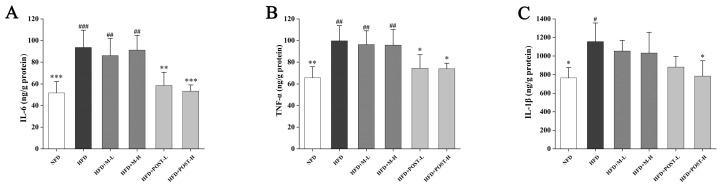
POST reduces hepatic inflammation in HFD mice. The concentration of cytokines in the liver, (**A**) IL-6, (**B**) TNF-α, (**C**) IL-1β. Compared to HFD, * *p* < 0.05, ** *p* < 0.01, and *** *p* < 0.001; Compared to NFD, # *p* < 0.05, ## *p* < 0.01, and ### *p* < 0.001.

Inflammation is closely related to fat accumulation; therefore, we evaluated changes in inflammation-related cytokines in the liver. Compared with the NFD group, the HFD group had significantly up-regulated pro-inflammatory cytokines (IL-6, *p* < 0.001; TNF-α, *p* = 0.0018; IL-1β, p = 0.0096; Figure 4A–C). M failed to improve liver inflammation, whereas POST significantly inhibited the elevation of IL-6 (HFD + POST-L vs. HFD: decreased 0.37-fold, *p* = 0.0029; HFD + POST-H vs. HFD: decreased 0.43-fold, *p* < 0.001) and TNF-α (HFD + POST-L vs. HFD: decreased 0.25-fold, *p* = 0.0282; HFD + POST-H vs. HFD: decreased 0.26-fold, *p* = 0.0237) levels in HFD mice; additionally, a POST-H intervention significantly decreased the IL-1β (decreased 0.32-fold, *p* = 0.0149) levels (Figure 4A–C).

### 2.5. POST Altered the Gut Microbiota Composition

**Figure 5:**
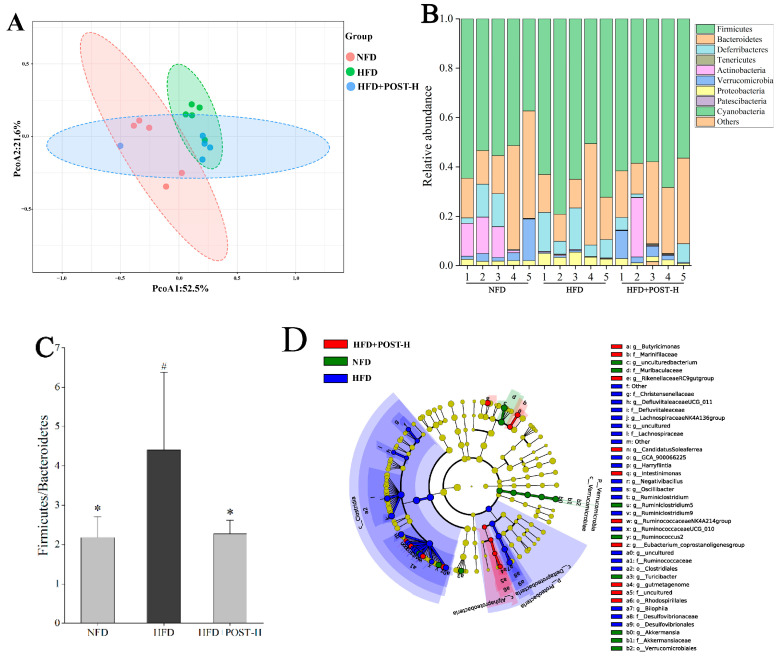

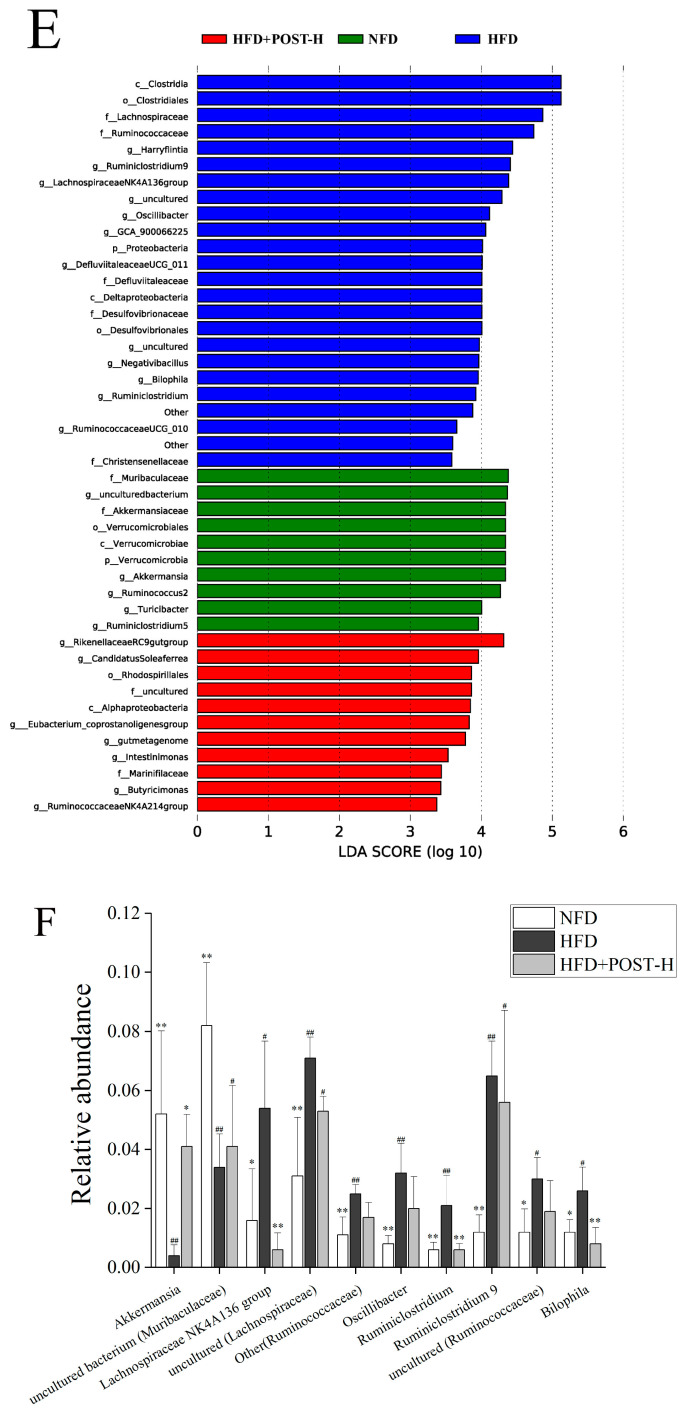
POST regulates gut microbiota. (**A**) PCoA analysis (PCoA1 = 52.5%, PCoA2 = 21.6%). (**B**) phylum-level microbial relative abundance (**C**) The value of F/B. (**D**) Cladograms for LEfSe analysis. (**E**) LDA scores. (**F**) The top 10 differential genera in relative abundance. Compared to HFD, * *p* < 0.05, and ** *p* < 0.01; Compared to NFD, # *p* < 0.05, and ## *p* < 0.01.

The regulatory effect of POST on the richness and diversity of gut microbiota was evaluated using high-throughput sequencing. The results of Alpha diversity showed that there was no significant difference in the shannon index and chao1 index between groups (Supplementary Figure S1). The detection results were analyzed for beta diversity using principal coordinates analysis (PCoA), and although the NFD group and HFD group were separated, they were clustered with the POST-H treatment group (Figure 5A). This indicates that the differences in species richness and species correlation in the gut between HFD and NFD mice were reduced after the POST-H treatment. *Firmicutes* and *Bacteroidetes* were the most prevalent phyla across all animal groups; other dominant phyla included *Deferribacteria*, *Proteobacteria*, *Verrucomicrobia*, and *Actinobacteria* (Figure 5B). HFD increased the ratio of *Firmicutes* to *Bacteroidetes* (F/B), whereas the POST-H supplementation significantly suppressed this change (Figure 5C). The LEfSe analysis bacterial group difference threshold was set as an LDA score > 3.0, α < 0.05, and a total of 26 genera were detected to have differences between groups (Figure 5D,E), of which 15 genera belonged to *Ruminococcaceae*. HFD altered the abundance of different genera, some of which were restored after the POST-H intervention. In our study, we selected the top 10 highly-abundant differential genera to analyze their expression in each group. The results showed that HFD significantly increased the relative abundance of *uncultured (Lachnospiraceae)*, *Lachnospiraceae NK4A136 group*, *Other (Ruminococcaceae)*, *Oscillibacter*, *Ruminiclostridium*, *Ruminiclostridium* 9, *uncultured (Ruminococcaceae)*, and *Bilophila*, but significantly decreased the relative abundance of *uncultured bacterium (Muribaculaceae)* and *Akkermansia* (Figure 5F). However, the supplementation with POST-H reversed the HFD-induced changes in genus abundance, significantly reducing *Lachnospiraceae NK4A136 group*, *Ruminiclostridium* and *Bilophila* while significantly increasing *Akkermansia* (Figure 5F). 

### 2.6. Correlation Analysis and Prediction of Microbial Metabolic Function

**Figure 6:**
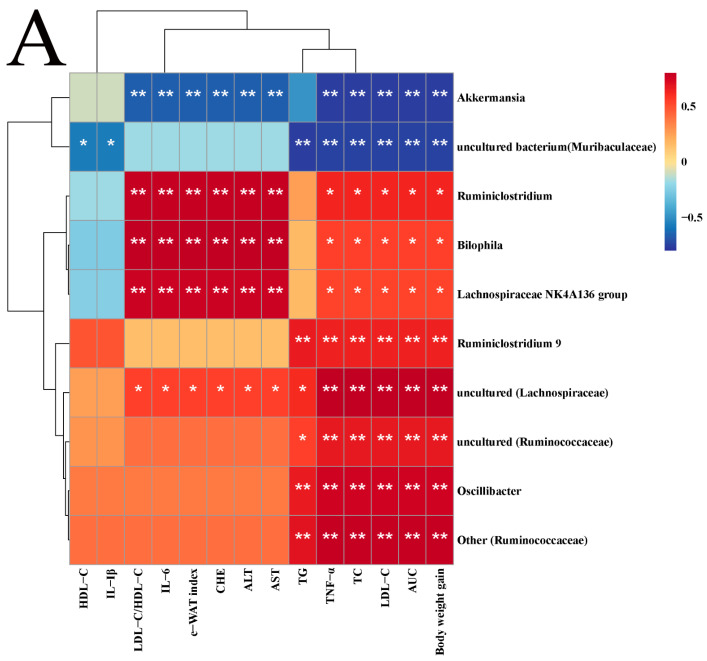

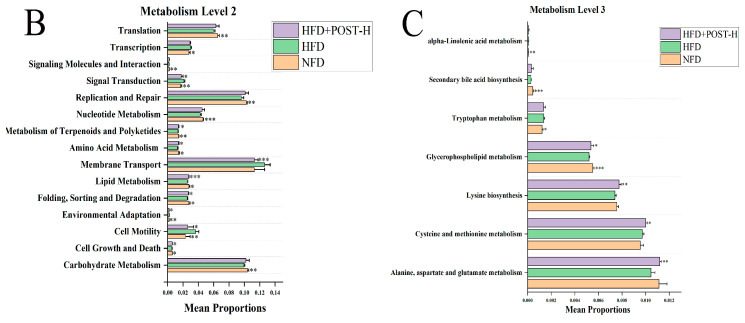
Correlation analysis (**A**) and microbial metabolic function of KEGG at level 2 (**B**) and level 3 (**C**). Correlation, blue for negative and red for positive, the asterisk represents the significance of the correlation value, * *p* < 0.05, ** *p* < 0.01. Compared to HFD, * *p* < 0.05, ** *p* < 0.01, and *** *p* < 0.001.

Spearman analysis was used to determine the associations between different genera in terms of high abundances and NAFLD symptoms (Figure 6A). *Lachnospiraceae NK4A136 group*, *Ruminiclostridium*, and *Bilophila* had significantly negative correlations with all lipid metabolism parameters except TG, HDL-C, and IL-1β. *Ruminiclostridium* 9, *uncultured (Ruminococcaceae)*, *Oscillibacter*, and *Other (Ruminococcaceae)* had significantly positive correlations with weight gain, AUC, TNF-α, and serum lipid indices other than HDL-C. *Uncultured (Lachnospiraceae)* had a significantly positive correlation with all indices except HDL-C and IL-1β, but *Akkermansia* and *uncultured (Lachnospiraceae)* were completely opposite. *Uncultured bacterium (Muribaculaceae)* was negatively associated with blood lipids, AUC, weight gain, and the IL-1β and TNF-α indices.

The gut microbiota metabolism of mice was predicted by PICRUSt2. Compared with the HFD group, lipid metabolism, amino acid metabolism and other metabolic pathways were significantly enriched in HFD + POST-H and NFD groups, indicating that POST supplementation can partially reverse the gut microbiota function of HFD mice (Figure 6B). We further studied the tertiary KEGG pathway of lipid metabolism and amino acid metabolism (Figure 6C), and the results showed that POST-H supplementation significantly promoted the relevant metabolic pathways in HFD mice, including alanine, aspartate and glutamate metabolism, cysteine and methionine metabolism, lysine biosynthesis and glycerophospholipid metabolism.

### 2.7. POST Modulated the Hepatic Metabolome in HFD Mice

**Figure 7:**
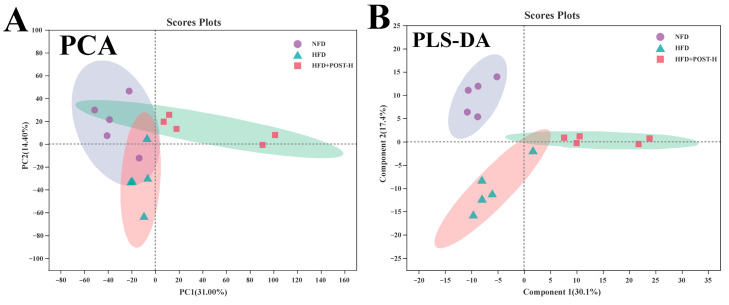

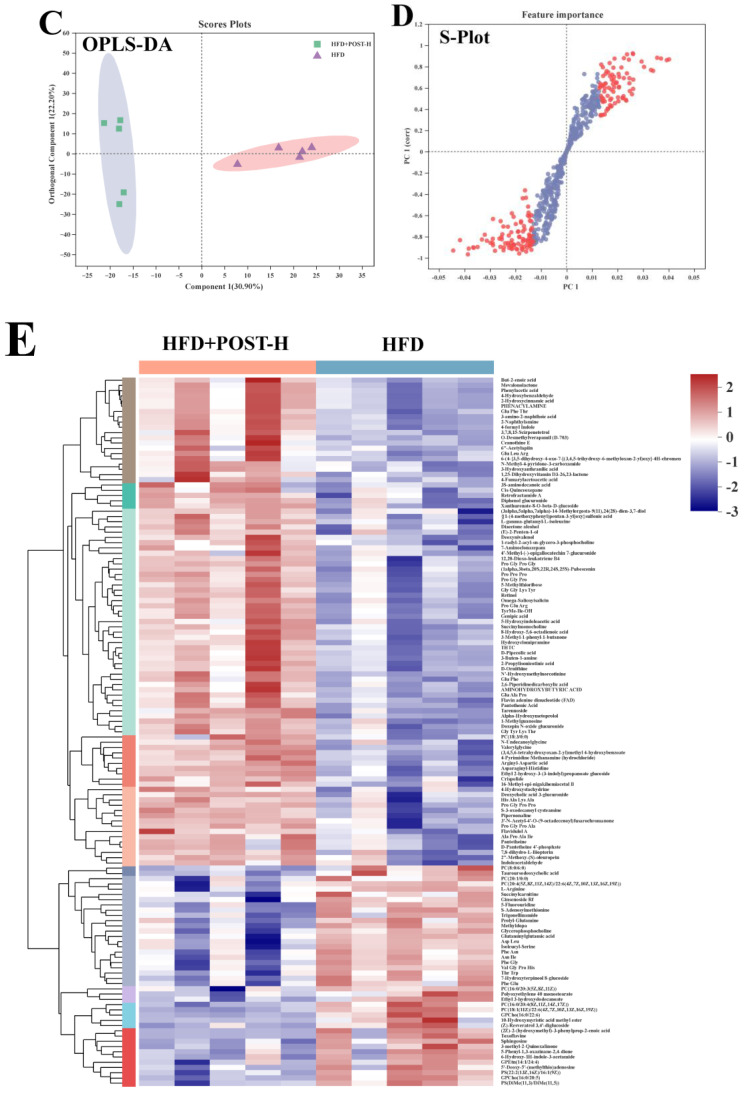

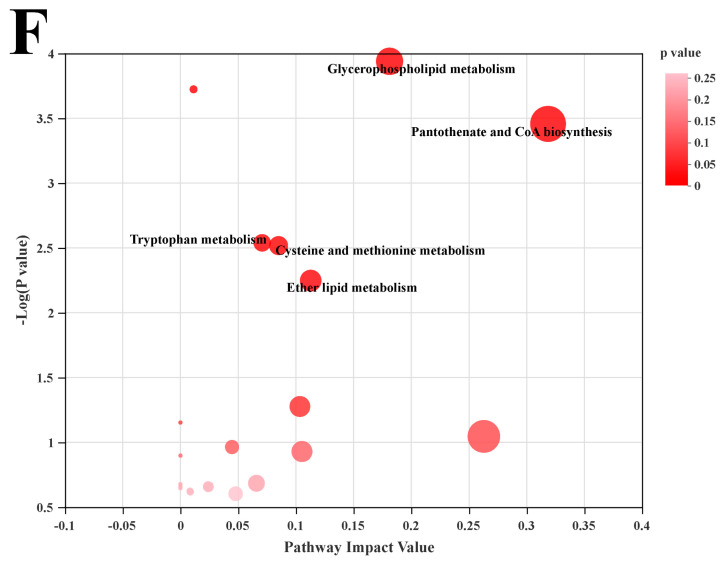
POST regulates hepatic metabolism (ESI+). (**A**) PCA score of the NFD, HFD and HFD + POST-H. (**B**) PLS-DA score of the NFD, HFD and HFD + POST-H. (**C**) OPLS-DA score of the HFD and HFD + POST-H. (**D**) S-loading plot based on OPLS-DA analysis of the HFD and HFD + POST-H. The red points indicate that these metabolites have a VIP value of 1 or greater, and the blue points indicate that these metabolites have a VIP value of less than 1. (**E**) Heat map of relative abundance of differential metabolites in liver between HFD and HFD + POST-H. (**F**) The effects of POST-H on liver metabolic pathways were analyzed based on KEGG.

**Figure 8:**
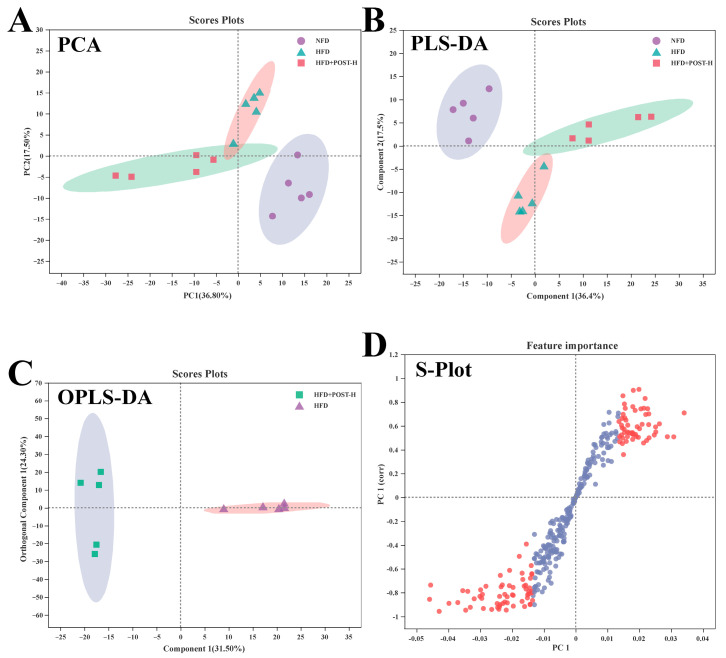

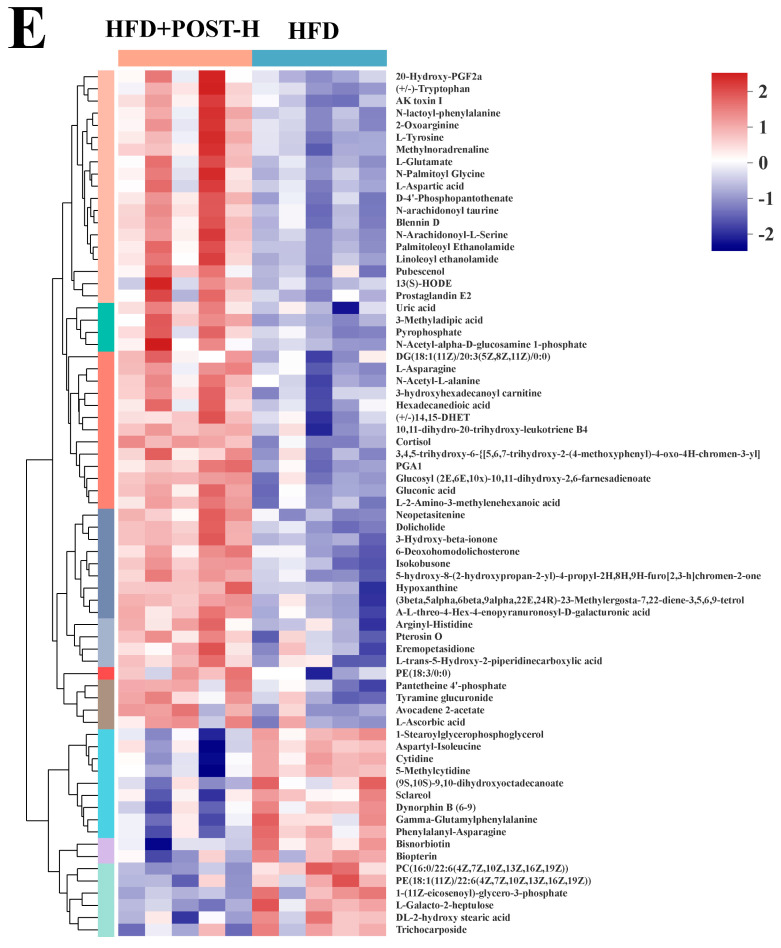

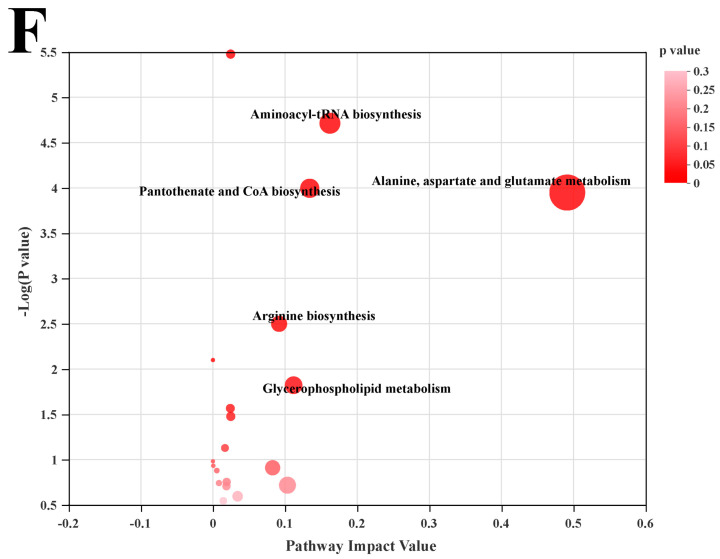
POST regulates hepatic metabolism (ESI-). (**A**) PCA score of the NFD, HFD and HFD + POST-H. (**B**) PLS-DA score of the NFD, HFD and HFD + POST-H. (**C**) OPLS-DA score of the HFD and HFD + POST-H. (**D**) S-loading plot based on OPLS-DA analysis of the HFD and HFD + POST-H. The red points indicate that these metabolites have a VIP value of 1 or greater, and the blue points indicate that these metabolites have a VIP value of less than 1. (**E**) Heat map of relative abundance of differential metabolites in liver between HFD and HFD + POST-H. (**F**) The effects of POST-H on liver metabolic pathways were analyzed based on KEGG.

We used LC-MS metabolomic analysis to detect the metabolic profile of the mouse liver and further explored the mechanism of action of POST. Based on the PCA and PLS-DA analysis results, the hepatic metabolites in the NFD, HFD, and HFD + POST-H groups were clearly differentiated, and the POST-H intervention partially reversed the metabolite changes caused by HFD (Figure 7A,B and Figure 8A,B). The score plot of the OPLS-DA model illustrated a clear separation between the HFD and HFD + POST-H groups (Figure 7C and Figure 8C). The metabolites far from the center in the loading graph were liver metabolites (potential biomarkers) that were markedly altered after the HFD + POST-H intervention (Figure 7D and Figure 8D). In this study, potential biomarker metabolites were screened based on the VIP value (>1.0) and *p*-value (<0.05), which explain the differences between the HFD + POST-H and HFD groups. In the liver, 206 potential biomarkers were identified in the 2 ion modes. After the POST-H intervention in HFD mice, the contents of 93 metabolites significantly increased, 42 metabolites significantly decreased in the positive ion mode, 54 metabolites increased markedly, and 17 metabolites decreased markedly in the negative ion mode (Figure 7E and Figure 8E). We performed a KEGG analysis of the metabolic pathways of hepatic differential metabolites to gain insight into the effects of POST-H on HFD mice. The metabolic pathways significantly affected by POST-H were selected according to their impact value (>0.05) and *p*-value (<0.05). The results showed that POST-H significantly regulated glycerophospholipid metabolism, pantothenate and CoA biosynthesis, cysteine and methionine metabolism, tryptophan metabolism, alanine, aspartate, and glutamate metabolism, ether lipid metabolism, aminoacyl-tRNA biosynthesis, and arginine biosynthesis (Figure 7F and Figure 8F).

### 2.8. POST Regulated Hepatic Lipid Metabolism Gene Expression

**Figure 9:**
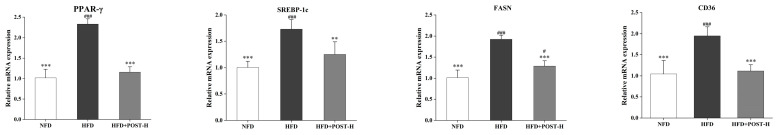

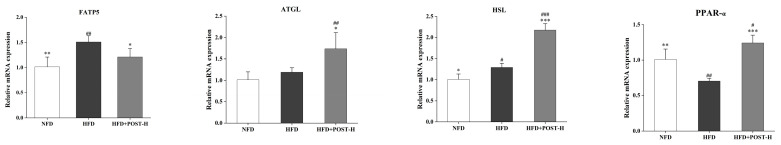
POST regulates hepatic lipid metabolism. Relative mRNA expression of PPAR-γ, SREBP-1c, FASN, CD36, FATP5, ATGL, HSL, and PPAR-α in the liver. Compared to HFD, * *p* < 0.05, ** *p* < 0.01, and *** *p* < 0.001; Compared to NFD, # *p* < 0.05, ## *p* < 0.01, and ### *p* < 0.001.

The mRNA expression of liver lipid metabolism genes was evaluated using qRT-PCR to clarify the possible mechanism underlying the effects of POST. In terms of gene expression, Figure 9 shows that HFD promotes lipid synthesis and transport in the mouse liver, such as PPAR-γ (*p* < 0.001), sterol regulatory element binding proteins 1c (SREBP-1c) (*p* < 0.001), fatty acid synthase coding gene (FASN; *p* < 0.001), fatty acid translocase CD36 (CD36) (*p* < 0.001), and fatty acid transport protein 5 (FATP5) (*p* = 0.0014), whereas inhibiting fatty acid oxidative consumption, such as PPAR-α (*p* = 0.0022). However, after the POST-H intervention in HFD mice, lipid synthesis and transport were inhibited (PPAR-γ: decreased 0.50-fold, *p* < 0.001; SREBP-1c: decreased 0.28-fold, *p* = 0.004; FASN: decreased 0.33-fold, *p* < 0.001; CD36: decreased 0.43-fold, *p* < 0.001; FATP5: decreased 0.20-fold, *p* = 0.0365), and fatty acid oxidation was restored (PPAR-α: increased 0.77-fold, *p* < 0.001). Notably, POST-H also substantially raised the mRNA expression of lipolytic enzymes, such as adipose triglyceride lipase (ATGL; increased 0.46-fold, *p* = 0.0133) and HSL (increased 0.68-fold, *p* < 0.001) in HFD mice.

## 3. Discussion

The symptoms of NAFLD include the excessive accumulation of fat in hepatocytes or steatosis, which is the first step in its pathogenesis [19]. Probiotics have long been recognized as a promising option for easing and/or preventing NAFLD [20]. In recent studies, postbiotics have also been identified as potential therapies for NAFLD [21]. The oral administration of *Lactobacillus plantarum* L-14-prepared postbiotics significantly inhibits adipogenesis in HFD mice, and this beneficial effect may be attributed to the exopolysaccharides of the postbiotics [22]. Postbiotics derived from *Akkermansia muciniphila* have been found to play a key role in regulating metabolic functions to prevent obesity [23]. In our study, we found that postbiotics prepared from *Lactobacillus paracasei* CCFM1224 can effectively prevent the development of NAFLD in HFD-fed mice, and this protective effect may be achieved by modulating the gut microbiome and liver metabolomics.

In our study, hepatic steatosis was induced in HFD-fed mice [24]. It was shown that POST considerably inhibited the body weight increase in mice fed an HFD and decreased fat accumulation in e-WAT compared with that in the HFD group. POST might inhibit white fat accumulation in mice by regulating the expression of lipid metabolism genes in e-WAT. In HFD mice, adipose tissue dysfunction promotes lipolysis and subsequent hyperlipidemia [25]. The POST intervention inhibited the HFD-induced elevation of serum lipids and liver enzymes caused by HFD, and these indicators clearly indicated changes in lipid metabolism and impairment of liver function [26]. In addition, POST increased glucose tolerance in HFD mice and effectively prevented IR, which is positively correlated with the occurrence of NAFLD [27]. The liver histological analysis also demonstrated that POST had a hepatoprotective effect, preventing fatty liver by inhibiting steatosis. The excessive accumulation of liver fat can cause lipotoxicity, resulting in hepatocyte damage and inflammation [28]. Therefore, chronic liver inflammation was present in NAFLD patients, and hepatocytes’ induction of pro-inflammatory cytokines by an excessive lipid content resulted in NAFLD [29]. Reducing inflammation may contribute to the amelioration of NAFLD, as evidenced by POST significantly inhibiting the HFD-induced elevation of hepatic inflammatory cytokines in this study. Studies have shown that POST can improve NAFLD symptoms in HFD mice, independent of the composition of the medium itself. However, the mechanism by which POST prevents NAFLD requires further investigation. 

Gut microbiota is closely associated with NAFLD [30]. A high *Firmicutes/Bacteroidetes* (F/B) ratio is a feature of the gut microbiota in obese animals [31], and the results of our study showed that a POST-H intervention substantially suppressed the HFD-induced elevation of the F/B ratio by reducing the *Firmicutes* abundance and increasing the *Bacteroidetes* abundance. Studies have shown that *Lachnospiraceae* is positively associated with the development of obesity and diabetes in mice [32]. We found that a POST-H restored the HFD-induced elevation in the *Lachnospiraceae NK4A136 group*, which was positively correlated with indicators that induce NAFLD. This is similar to the results of previous studies [33]. POST-H also suppressed the HFD-induced elevation in the relative abundance of *Bilophila* and other genera in *Ruminococcaceae*. *Bilophila* is thought to be positively associated with hepatic lipid accumulation and is enriched in the gut in HFD [34]. The occurrence of NAFLD is often accompanied by an increase in the abundance of *Ruminococcaceae* [35]. HFD can significantly reduce the relative abundance of *Akkermansia* [36], and our study yielded similar results, whereas POST-H intervention prevented this downregulation. Research has demonstrated that *Akkermansia* levels are negatively correlated with NAFLD development [37]. Therefore, POST may prevent NAFLD by restoring the gut microbiota imbalance caused by an HFD.

Metabolomics is a powerful method for the untargeted analysis of small molecules in tissue samples [38]. Our results suggest that POST alleviates NAFLD mainly by modulating metabolic pathways, such as glycerophospholipid and ether lipid metabolism, pantothenate and CoA biosynthesis, and amino acid metabolism. As an important component of mammalian cells, glycerophospholipids are involved in many cellular processes related to metabolic syndromes, such as molecular transport and protein function. Studies have shown that glycerophospholipid metabolism disorders disrupt the energy metabolism in liver cells [39]. NAFLD is often accompanied by abnormal hepatic PC or PE levels, and excessive PC or PE levels can cause energy metabolism disorders and cell damage [40]. POST-H intervention significantly reduced the PC and PE content in the hepatocytes of HFD mice and regulated glycerophospholipid metabolic disorders. Ether lipid metabolism disorders are closely related to metabolic diseases [41]. Ether phospholipids are mainly produced in the liver, and fatty acids synthesized by ether lipids are derived from FASN-mediated de novo lipogenesis [42]. Decreased levels of endogenous plasmalogens are associated with the impaired expression of PPAR-α [43]. POST-H markedly increased the levels of plasmalogens (such as 1-radyl-2-acyl-sn-glycero-3-phosphocholine) in the hepatic ether lipid metabolism pathway in HFD mice and regulated lipid metabolism disorders. The biosynthetic pathways of pantothenic acid and CoA play important roles in various physiological and pathological cellular processes [44]. Pantothenic acid is a key precursor of CoA and is involved in the synthesis of key enzymes of the TCA cycle in the body, and a lack of pantothenic acid results in reduced ATP synthesis [45]. POST-H significantly increased the content of related metabolites in pantothenate and CoA biosynthesis, such as pantothenic acid and D-4′-Phosphopantothenate, and promoted energy metabolism in the mouse liver. The POST-H intervention increased the levels of hepatic tryptophan metabolites such as 5-Hydroxyindoleacetic acid, indoleacetaldehyde, and 3-Hydroxyanthranilic acid, which have significant anti-inflammatory effects [46]. Similarly, alanine, aspartate, and glutamate metabolism, which are central to glutamate metabolism, are closely related to the occurrence of inflammation [47]. Methionine can promote lipoprotein synthesis, which is conducive to the transport of fat out of the liver, thereby relieving fatty liver [48]. Studies have shown that L-cysteine can reduce lipid levels in rat serum and liver [49]. POST-H also decreased the S-Adenosylmethionine and 5′′-Deoxy-5′′- (methylthio) adenosine content in the cysteine and methionine metabolism pathways, suggesting that it may prevent NAFLD by reducing the hepatic metabolic depletion of cysteine and methionine. Therefore, we speculated that POST might prevent NAFLD by regulating liver metabolism, which is also consistent with the partial prediction of intestinal microbiota in lipid and amino acid metabolism.

Changes in liver metabolism are closely related to liver gene expression [50]. Hepatocyte steatosis is typically caused by increased fatty acid synthesis and decreased fatty acid oxidation [51]. Therefore, we investigated the expression of the genes associated with hepatic lipid metabolism. In the development of NAFLD, PPAR-γ can stimulate the expression of downstream FASN by regulating SREBP-1c [52], while SREBP-1c has the ability to upregulate the gene expression of de novo adipose synthase FASN and is a major regulator of hepatic lipogenesis [53]. CD36 is involved in fatty acid transmembrane transport, uptake and other metabolic processes and can mediate metabolic dysregulation of liver inflammation [54]. FATP5 is mainly distributed in liver tissue and can increase the absorption of long-chain fatty acids [55]. ATGL is the first step in regulating lipid lipolysis, after which HSL facilitates the removal of excess fatty acids [56,57]. The overexpression of HSL or ATGL in the liver can promote fatty acid oxidation and improve hepatic steatosis, which is related to the activation of the PPAR-α [58]. PPAR-α is highly expressed in the normal liver, and its expression gradually decreases with the accumulation of liver lipids [59]. In our study, POST inhibited the expression of PPAR-γ, SREBP1c, FASN, CD36 and FATP5 while promoting the expression of HSL, ATGL and PPAR-α in HFD mice. Therefore, we speculated that the mechanism by which POST prevents NAFLD is closely related to a decrease in fatty acid synthesis and absorption in hepatocytes and an increase in fatty acid oxidation.

## 4. Materials and Methods

### 4.1. Postbiotics Preparation

*Lactobacillus paracasei* CCFM1224 was obtained from the bacterial bank of the Food Biotechnology Research Center of Jiangnan University. *Lactobacillus paracasei* CCFM1224 was activated to the third generation in MRS medium. The activated seed solution was inoculated at 2% into the postbiotic preparation medium (each liter of water contained 60 g glucose, 20 g casein peptone, 1 g yeast extract, 0.35 g MgSO_4_·7H_2_O, 0.1 g MnSO_4_·H_2_O, and 2.6 g K_2_HPO_4_·3H_2_O) and was cultured for 12 h. The resulting fermentation broth was inoculated at 5% into the postbiotic preparation medium and cultured for 30 h. The fermentation broth was heat-treated (65 °C, 30 min), sonicated (crushing power 60%, total crushing time 15 min, intermittent time 4 s), and freeze-dried to obtain postbiotic. The resulting postbiotic (POST) and postbiotic preparation media (M) were lyophilized for later use.

### 4.2. Animal Experiments

The Experimental Animal Ethics Committee of Jiangnan University approved this study (qualified number: JN. No 20210330c1250715(056)). Male C57BL/6N mice were acquired from Vital River Co., Ltd. (Beijing, China). The mice were acclimated for 1 week in an SPF-rated facility with unrestricted access to water and food. Thirty mice were randomly divided into 6 groups, with 5 mice in each group. Mice in each group were fed either a normal-fat diet (NFD, TP23302, TROPHIC, Nantong, China) or a high-fat diet (HFD, TP23300, TROPHIC, Nantong, China). The detailed grouping was as follows: (1) NFD: an NFD was provided to mice; (2) HFD: an HFD was provided to mice; (3,4) HFD + M-L, HFD + M-H: mice were fed an HFD, and there was M intervention (M-L: 200 mg/kg/day, M-H: 800 mg/kg/day); (5,6) HFD + POST-L, HFD + POST-H: mice were fed an HFD, and there was POST intervention (POST-L: 200 mg/kg/day, POST-H: 800 mg/kg/day). POST and M were administered to mice by gavage at the appropriate doses dissolved in 0.2 mL of saline once daily for 12 weeks, and the NFD and HFD groups were given the same amount of normal saline. The mice were weighed weekly during the experiment. Blood was drawn from the eyes, while the serum was obtained by centrifugation after standing and was stored at -80 °C. The epididymal white fat (e-WAT) was weighed after dissection.

### 4.3. Oral Glucose Tolerance Test (OGTT)

Before the mice were euthanized, an OGTT was conducted on all the animals. After a 12-h fast, glucose was administered by gavage at a dose of 2 mg/g mouse weight. Six time points were set for blood glucose measurements: before gavage and 15, 30, 60, 90, and 120 min after gavage. Accu-Chek Active test strips (Roche Diabetes Care, Mannheim, Germany) were used to test fresh blood samples from the tail veins of the mice. GraphPad Prism 6 was used to determine the area under the curve (AUC) for the OGTTs.

### 4.4. Serum Biochemical Index Assays

A Mindray biochemical analyzer (Mindray, Shenzhen, China) was used to determine the levels of serum biochemical markers associated with NAFLD, including total cholesterol (TC), triacylglycerol (TG), low-density lipoprotein cholesterol (LDL-C), high-density lipoprotein cholesterol (HDL-C), alanine aminotransferase (ALT), aspartate aminotransferase (AST), and cholinesterase (CHE).

### 4.5. Histological Analysis

Parts of the liver tissue and epididymal adipose tissue were cut and fixed in neutral paraformaldehyde during dissection. The fixed tissues were dehydrated and embedded in paraffin. Hematoxylin and eosin (H&E) were used to stain the tissue segments. Fresh liver tissue was quickly frozen and sectioned and stained with Oil Red O. The sections were scanned using a digital slide scanner (Pannoramic MIDI II, 3DHISTECH Ltd., Budapest, Hungary). The pathological scores of hepatic steatosis and inflammation were performed according to the study of Liang et al. [60], and the specific criteria are shown in Supplementary Table S1.

### 4.6. Liver Inflammatory Cytokine Assay

Hepatocyte cytokines, including IL-6 (DY406), TNF-α (DY410) and IL-1β (DY401), were measured using a commercial mouse ELISA kit (R&D Systems, Minneapolis, MN, USA), according to the manufacturer’s instructions.

### 4.7. Gut Microbiota Sequencing

Feces were collected before the mice were euthanized. Total DNA from fecal microorganisms was isolated using a Fast DNA Stool Kit (M.P. Biomedicals, Irvine, CA, USA). The V3-V4 region of the 16S rRNA gene was amplified using the primers 341F and 806R. Nucleic acid gel electrophoresis was used to purify the PCR amplification products, and a PCR purification kit (TIANgel Mini Purification Kit; TIANGEN, Beijing, China) was used to purify the amplicons on the gels. Sequencing was performed using an Illumina MiSeq PE300 platform (Illumina, San Diego, CA, USA). Quantitative Insights Into Microbial Ecology2 (QIIME2) [61] and Tax4Fun2 were used to determine the makeup of the microbes [62]. The SILVA database was used for the alignment and taxonomical assignment of representative sequences for each OTU. The alpha and beta diversity were analyzed using q2-diversity to assess the diversity of the samples. Alpha diversity was characterized by the shannon and chao1 diversity index, and beta diversity was characterized by PCoA based on the Bray–Curtis distance between samples.

### 4.8. Liver Metabolomics

The internal standard L-2-chlorophenylalanine was added to a solution with a methanol/water volume of 4:1 at 0.02 mg/mL to prepare an extract solution. Liver samples (50 mg) were weighed, mixed with 400 µL of the extract solution, homogenized at low temperature, and centrifuged after low-temperature ultrasonic extraction. The supernatant was used for LC-MS analysis. Twenty microliters of each sample were mixed to prepare a quality control sample. The instrument platform for LC-MS analysis was the UHPLC-Q Exactive HF-X system (Thermo Scientific), and the chromatographic column was an ACQUITY UPLC HSS T3 (Waters Corporation, Milford, MA USA). Mobile phase A consisted of 95% water and 5% acetonitrile; mobile phase B consisted of 47.5% acetonitrile, 47.5% isopropanol, and 5% water; and both mobile phases A and B contained 0.1% formic acid. The elution gradients used for the analysis are listed in Supplementary Table S2. Mass spectral signals of the samples were collected using positive and negative ion scans. The mass spectrometry parameters are listed in Supplementary Table S3. Raw data were processed using Progenesis QI (Waters Corporation, Milford, MA, USA), and mass spectral information was then matched against a metabolic database to identify metabolites.

### 4.9. Quantitative Reverse Transcription PCR (qRT-PCR)

According to the product manual, total RNA was isolated from the liver using a UNlQ-10 Column Trizol Total RNA Isolation Kit (B511321, Sangon Biotech Co., Ltd., Shanghai, China) and complementary DNA was produced by HiScript^®^ III RT SuperMix for qPCR (+gDNA wiper) (R323, Vazyme Biotech Co., Ltd., Nanjing, China). The BioRad-CFX384 Touch thermocycler (Bio-Rad, Hercules, CA, USA) was used to perform quantitative real-time polymerase chain reaction (qPCR) using ChamQ Universal SYBR qPCR Master Mix (Q711, Vazyme Biotech Co., Ltd., Nanjing, China). The primers used for qRT-PCR are listed in Supplementary Table S4. The results were evaluated using the 2^−ΔΔCt^ method.

### 4.10. Statistical Analysis

The experimental results are expressed as mean ± standard deviation. The Shapiro–Wilk normality test was used to test the normality of the samples. A 1-way ANOVA was used for statistical analysis, and the significance of differences between groups was determined using Tukey’s multiple comparison test. A *p*-value < 0.05 indicates a statistically significant difference. Compared to HFD, * *p* < 0.05, ** *p* < 0.01, and *** *p* < 0.001; Compared to NFD, # *p* < 0.05, ## *p* < 0.01, and ### *p* < 0.001.

## 5. Conclusions

This study demonstrated that the POST treatment ameliorated HFD-induced NAFLD by inhibiting body weight gain and epididymal white fat accumulation, improving serum biochemical indicators, and preventing hepatic steatosis and inflammation. Gut microbiome and liver metabolomics revealed the key microbiota and important metabolic pathways and biomarkers associated with NAFLD. These results suggest that POST alters the gut microbiota composition, improves liver metabolism, and regulates the expression of hepatic lipid metabolism-correlated genes. These findings provide a reference for identifying postbiotics that can ameliorate NAFLD. However, the key functional components and mechanisms of action of POST in improving NAFLD require further investigation by targeting metabolomics and humanized mouse models of gut microbiota.

## Figures and Tables

**Table 1:** Steatosis and inflammation scores of livers.

Groups	NFD	HFD	HFD + M-L	HFD + M-H	HFD + POST-L	HFD + POST-H
Macrovesicular steatosis score	0	2	1	2	0	0
Microvesicular steatosis score	0	3	3	3	1	0
Hypertrophy score	0	2	2	1	0	0
Total steatosis score	0	7	6	6	1	0
Inflammatory score	0	1	1	1	0	0

## Data Availability

All data presented in this study are available in the main body of the manuscript.

## Supplementary Materials

The following supporting information can be downloaded at: https://www.mdpi.com/article/10.3390/ijms232113522/s1.
