# Supplementary material for: Postbiotics Prepared Using Lactobacillus paracasei CCFM1224 Prevent Nonalcoholic Fatty Liver Disease by Modulating the Gut Microbiota and Liver Metabolism
Source: Int J Mol Sci. 2022 Nov 4;23(21):13522. doi: 10.3390/ijms232113522 (PMC9653709; doi:10.3390/ijms232113522)

Supplementary Table S1. Grading system for NAFLD

Histological feature	Score			
	0	1	2	3
Steatosis:	<5%	5-33%	33-66%	>66%
Macrovesicular steatosis	<5%	5-33%	33-66%	>66%
Microvesicular steatosis	<5%	5-33%	33-66%	>66%
Hypertophy	<5%	5-33%	33-66%	>66%
Inflammation:				
Number of inflammatory foci/field	<0.5	0.5-1.0	1.0-2.0	>2.0

Supplementary Table S2. Mobile phase elution gradient

Time (min)	Flow rate (mL/min)	A (%)	B (%)
0	0.4	100	0
3.5	0.4	75.5	24.5
5	0.4	35	65
5.5	0.4	0	100
7.4	0.6	0	100
7.6	0.6	48.5	51.5
7.8	0.5	100	0
9	0.4	100	0
10	0.4	100	0

Supplementary Table S3. Mass spectrometry parameters

Description	Parameters
Scan type (m/z)	70-1050
Sheath gas flow rate (arb)	50
Aux gas flow rate (arb)	13
Heater temp (℃)	425
Capillary temp (℃)	325
Spray voltage (+) (V)	3500
Spray voltage (-) (V)	-3500
S-Lens RF Level	50
Normalized collision energy (eV)	20,40,60
Resolution (Full MS)	60000
Resolution (MS2)	7500

Supplementary Table S4. Primer sequences for quantitative real-time PCR of hepatic genes involved in lipid metabolism

Primers	Forward Sequences (5′→3′)	Reverse Sequences (5′→3′)
PPAR-γ	TCGCTGATGCACTGCCTATG	GAGAGGTCCACAGAGCTGATT
SREBP-1c	TGACCCGGCTATTCCGTGA	CTGGGCTGAGCAATACAGTTC
FASN	GGAGGTGGTGATAGCCGGTAT	TGGGTAATCCATAGAGCCCAG
ATGL	GGATGGCGGCATTTCAGACA	CAAAGGGTTGGGTTGGTTCAG
HSL	CCAGCCTGAGGGCTTACTG	CTCCATTGACTGTGACATCTCG
PPAR-α	AGAGCCCCATCTGTCCTCTC	ACTGGTAGTCTGCAAAACCAAA
CD36	ATGGGCTGTGATCGGAACTG	GTCTTCCCAATAAGCATGTCTCC
FATP5	CTACGCTGGCTGCATATAGATG	CCACAAAGGTCTCTGGAGGAT

Supplementary Figure S1. Alpha diversity indices of gut microbiota

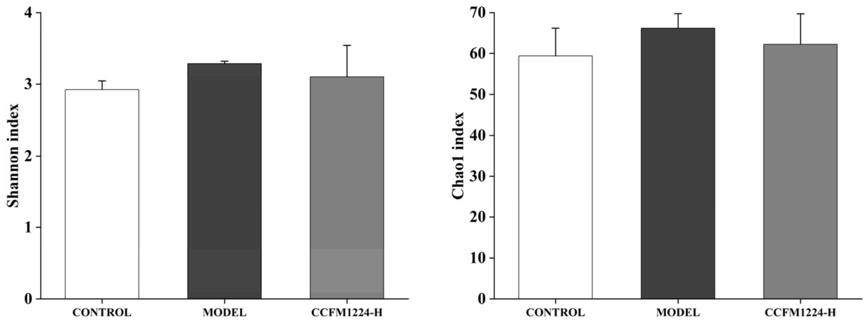

Supplement: Supplementary file 1 [file ijms-23-13522-s001.zip › ijms-1963357-supplementary.pdf]
